# Antibacterial effect of copper-bearing titanium alloy (Ti-Cu) against *Streptococcus mutans* and *Porphyromonas gingivalis*

**DOI:** 10.1038/srep29985

**Published:** 2016-07-26

**Authors:** Rui Liu, Kaveh Memarzadeh, Bei Chang, Yumei Zhang, Zheng Ma, Robert P. Allaker, Ling Ren, Ke Yang

**Affiliations:** 1 Northeastern University, 3-11 Wenhua Road, Shenyang 110819, China; 2 Institute of Metal Research, Chinese Academy of Sciences, 72 Wenhua Road, Shenyang 110016, China; 3 Institute of Dentistry, Barts and The London School of Medicine & Dentistry, Queen Mary University of London, Newark Street, London E1 2AT UK; 4 State Key Laboratory of Military Stomatology, Department of Prosthetic Dentistry, School of Stomatology, The Fourth Military Medical University, Xi’an 710032, China

## Abstract

Formation of bacterial biofilms on dental implant material surfaces (titanium) may lead to the development of peri-implant diseases influencing the long term success of dental implants. In this study, a novel Cu-bearing titanium alloy (Ti-Cu) was designed and fabricated in order to efficiently kill bacteria and discourage formation of biofilms, and then inhibit bacterial infection and prevent implant failure, in comparison with pure Ti. Results from biofilm based gene expression studies, biofilm growth observation, bacterial viability measurements and morphological examination of bacteria, revealed antimicrobial/antibiofilm activities of Ti-Cu alloy against the oral specific bacterial species, *Streptococcus mutans* and *Porphyromonas gingivalis*. Proliferation and adhesion assays with mesenchymal stem cells, and measurement of the mean daily amount of Cu ion release demonstrated Ti-Cu alloy to be biocompatible. In conclusion, Ti-Cu alloy is a promising dental implant material with antimicrobial/antibiofilm activities and acceptable biocompatibility.

Commercially pure titanium (Ti) is increasingly becoming the preferred option for replacing missing teeth, and occupies the largest market share in the oral metal materials/implants sector^1^. As regards bacterial diversity in an individual oral cavity this is represented by approximately 500 species, which can readily colonize different types of surfaces including teeth, prosthetic devices and dental implants^2^,^3^,^4^. The formation and maturation of biofilms on dental implant material surfaces may lead to the development of peri-implant diseases, such as peri-implant mucositis or peri-implantitis, influencing the long term success of the teeth implant^5^,^6^,^7^. For this reason, it is crucial to efficiently kill bacteria and discourage formation of biofilms on implant surfaces in order to inhibit such bacterial infection and prevent the failure of implant.

Although various measures, such as thorough disinfection and stringent aseptic surgical protocols, have been used to mitigate bacterial contamination, infection can often occur following surgery^8^. This is typically characterized as bacterial colonization and biofilm formation on oral surfaces including dental implants. Bacterial attachment on enamel and dentine starts with the recognition of salivary pellicle receptors by the initial colonizing bacteria on tooth surfaces^9^. The extracellular polymeric substance (EPS) forms on bacterial surfaces and the salivary pellicle provides binding sites for microorganisms, and subsequently the exopolysaccharide in the biofilm increases biofilm stability and structural integrity. Consequently, the biofilm can provide protection to the bacteria^10^. In the biofilm formed on oral implants, *Porphyromonas gingivalis* and *Streptococcus mutans* are two of the significant species, which have pronounced influence on promoting biofilm formation, inflammatory response and any bone defects^11^,^12^,^13^.

After biofilm formation on the surface of a dental implant, the removal and killing of bacteria remain a challenge despite the use of antibiotic therapy^4^. The emergence of resistant strains is an additional issue when antibiotics are administered^12^. To date, no treatment can guarantee rapid and complete elimination of the biofilm or prevention of secondary infection, and therefore long-term clinical success becomes dependent upon the antimicrobial properties of the employed dental materials^9^. Surface modification, such as incorporating antibacterial agents into implant materials, is a common method to provide antibacterial properties^4^,^5^,^6^,^7^,^8^. Mei *et al.* modified titanium surfaces using graded silver incorporation into titania nanotubes (NT-Ag), and the results indicated that NT-Ag enhanced antibacterial properties against *Porphyromonas gingivalis* and *Aggregatibacter actinomycetemcomitans* with satisfactory biocompatibility^13^. Previous studies have demonstrated the potential of antibacterial coatings to inhibit adhesion of bacteria and biofilm formation on oral implants. However, most of those attempts failed to deliver sustainable antibacterial effects, and the fast release of antimicrobial agents caused biosafety concerns^10^.

Copper (Cu) is a necessary trace element in the human body, and also a well-known alloying element with antimicrobial ability^14^. There have been studies as regards the application of the Cu element into stainless steel during the material making process to fabricate antibacterial stainless steels with excellent antibacterial properties^15^,^16^. Zhang *et al.* suggested that 304 type Cu-bearing stainless steel could kill *Porphyromonas gingivalis*^17^. Ma *et al.* reported a study on a newly developed Cu-bearing titanium alloy, Ti-6Al-4V-xCu (x = 1, 3, 5), which demonstrated an excellent antibacterial ability on *Escherichia coli (E. coli)* and *Staphylococcus aureus (S. aureus)* together with remarkable biocompatibility^14^,^18^,^19^. Shirai *et al.* also demonstrated that Ti-Cu alloy had antimicrobial activity and substantially reduced the incidence of pin tract infection^20^.

On the basis of the above studies, an appropriate amount of Cu was immobilized into pure titanium, to fabricate a Ti-Cu alloy to provide antibacterial properties for use as a dental material. In the present study, the antibacterial properties of the new implant material and the related antibacterial mechanism were investigated using real-time polymerase chain reaction (RT-PCR), scanning electron microscopy (SEM), confocal laser scanning microscopy (CLSM) and transmission electron microscopy (TEM). Additionally, the biocompatibility of the Ti-Cu alloy was evaluated *in vitro*.

## Results

### Biofilm based gene expression

**Figure 1:**
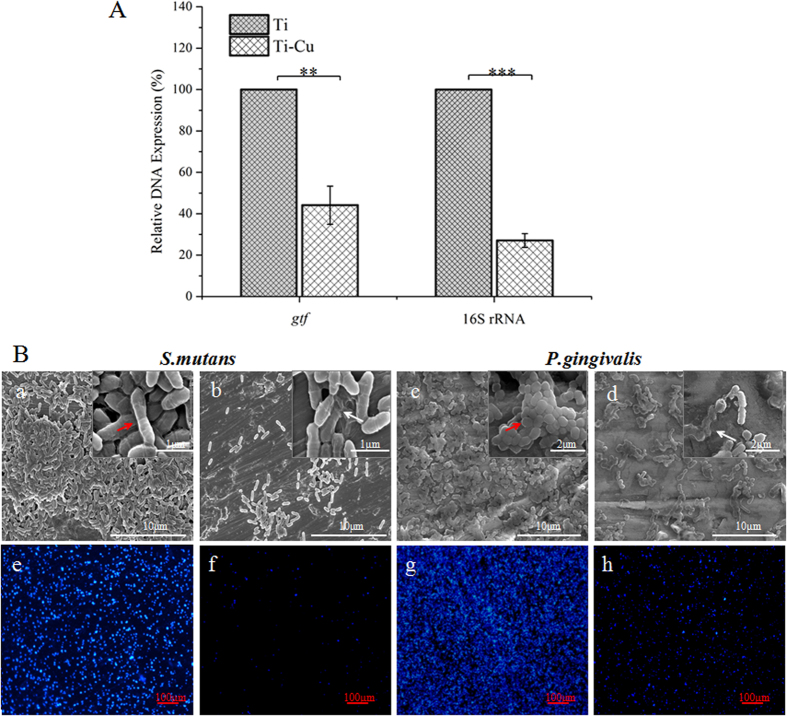
(**A**) Gene expressions of *P. gingivalis* (16s RNA) and *S. mutans* (glucosyltransferase, in short “*gtf* ”) in the biofilm, **P ≤ 0.01, ***P ≤ 0.001; (**B**) SEM micrographs and DAPI images of *S. mutans* and *P. gingivalis* on surfaces of Ti (a, c, e and g) and Ti-Cu alloy (b,d,f and h) after co-culture for 24 h.

RT-PCR experiments were performed to examine the effect of Ti-Cu alloy on the quantity of glucosyltransferase-genes (*gtfB* and *gtfC*) from *S. mutans* and normalizing internal standard gene (16S rRNA) from *P. gingivalis*, which were used to reflect the number of live bacteria on a surface. Independent amounts of DNA from early co-cultured bacteria were used to reveal the quantities of these genes with Ti or Ti-Cu alloy treatments. Ti-Cu alloy was found to have lower gene expressions of the two species. Specifically, the gene expression of *S. mutans* on Ti-Cu alloy was only 43.37% of that with Ti, whereas the gene expression of *P. gingivalis* on Ti-Cu alloy was only 26.91% of that with Ti (Fig. 1A), which suggested that Ti-Cu alloy is obviously antibacterial as compared to Ti.

### Biofilm observation

The obtained SEM micrographs of treated *S. mutans* and *P. gingivalis* at 24 h were in agreement with the RT-PCR results. *S. mutans* biofilms (Fig. 1b) on the surface of Ti-Cu alloy showed smaller clusters and were lower in population as compared with Ti (Fig. 1a). Similarly, when compared with Ti (Fig. 1c), *P. gingivalis* populations (Fig. 1d) on Ti-Cu alloy were lower and the biofilms were limited. Meanwhile, in terms of cell morphology, cells on Ti-Cu surface (Fig. 1b,d) showed extensive lack of abnormal shape and possible leaking out of the intracellular components causing cell damage (white arrows indicate representative damaged cells), whereas cells on Ti surface (Fig. 1a,c) possessed normal morphology (red arrows indicate representative cells).

### Bacterial viability within the biofilm

**Figure 2:**
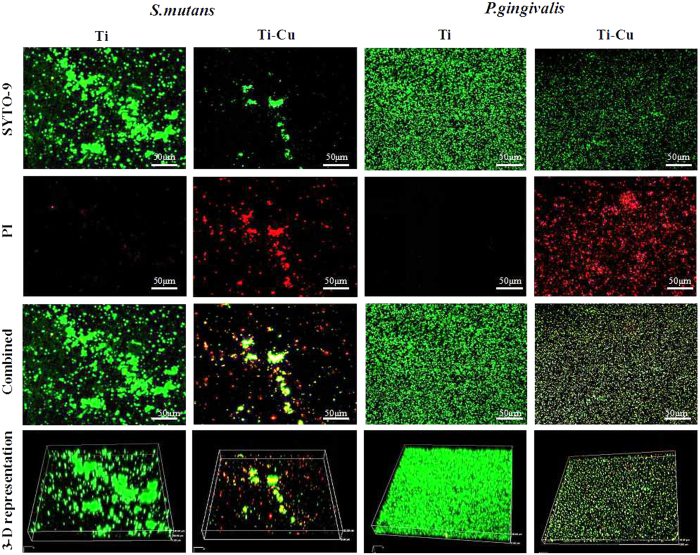
Fluorescent images and 3-D representations of *S. mutans* and *P. gingivalis* biofilms on surfaces of Ti and Ti-Cu alloy after incubation at 37 °C for 24 h, thickness of *S. mutans* biofilm on Ti is 40 μm, and not quantifiable on Ti-Cu alloy, while thickness of *P. gingivalis* biofilm on Ti is 36 μm, and that on Ti-Cu alloy is 18 μm.

To examine the viability of the biofilms on the samples, DAPI was used as a marker of nuclei in viable cells. A minor quantity of DAPI staining was observed on Ti-Cu surface when compared to Ti for both *S. mutans* (Fig. 1e,f) and *P. gingivalis* (Fig. 1g,h). These findings are in accordance with SEM observations. Biofilms were visualized using CLSM to observe the effects of Ti-Cu alloy and Ti using SYTO 9 (green) for staining viable and PI (red) for staining non-viable cells (Fig. 2). Compared with Ti, Ti-Cu alloy showed significantly less viable *S. mutans* cells. Furthermore, both *S. mutans* and *P. gingivalis* showed more scattered populations on Ti-Cu alloy. An NIS-Elements viewer software was used to perform the three-dimensional reconstruction, and the reconstruction graphs shown in Fig. 2 further supported the results from the CLSM micrographs. *S. mutans* biofilm grown on Ti exhibited a thickness of 40 μm, whereas no significant biofilm was formed on the Ti-Cu surface. Similarly, *P. gingivalis* biofilm grown on Ti exhibited a thickness of 36 μm, but showed half of this thickness (18 μm) on Ti-Cu surface.

### Morphological changes in *S. mutans* and *P. gingivalis*

**Figure 3:**
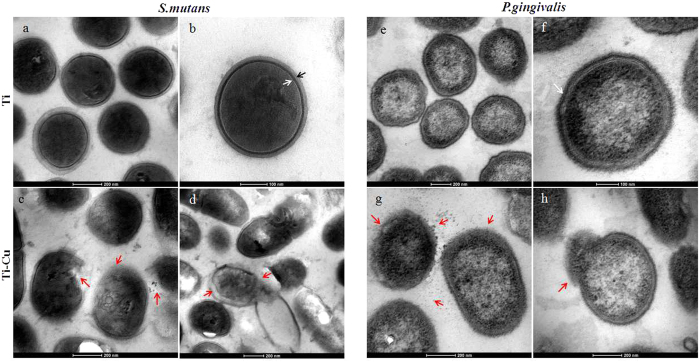
TEM micrographs of inner structures of *S. mutans* and *P. gingivalis*, (**a–d**) treated with Ti; (**e–h**) treated with Ti-Cu alloy. White and black arrows indicate peptidoglycan layer and cytoplasmic membrane, and red arrows indicate separation of the cell membrane from the cell wall and the release of cellular contents.

Figure 3 represents the TEM images of treated *S. mutans* and *P. gingivalis.* TEM was carried out to examine the morphological changes and intracellular modification of bacteria after interaction with Ti-Cu alloy. The bacteria cultured on Ti samples served for comparison, which showed a clear outline of the *S. mutans* cell wall and a peptidoglycan layer (Fig. 3a,b). It can be found that Ti-Cu alloy resulted in a disappearance of the peptidoglycan layer (Fig. 3e). This finding further indicates the destructive effect of the copper-containing titanium alloy on the bacterial cell wall that potentially leads to the separation of the cell membrane from the cell wall (Fig. 3f). TEM micrographs on *P. gingivalis* indicated an intact cell wall, membrane, a well-preserved peptidoglycan layer and cytoplasmic membrane (Fig. 3c,d) treated with Ti. However, obvious morphological changes were observed in the cells treated with Ti-Cu alloy, including compromised cell walls, reduction and uneveness in electron density in the cytoplasm (Fig. 3g,h). The localized separation of the cell membrane from the cell wall and the outflow of cytoplasm were also discerned.

### Cu^2+^ release

**Figure 4:**
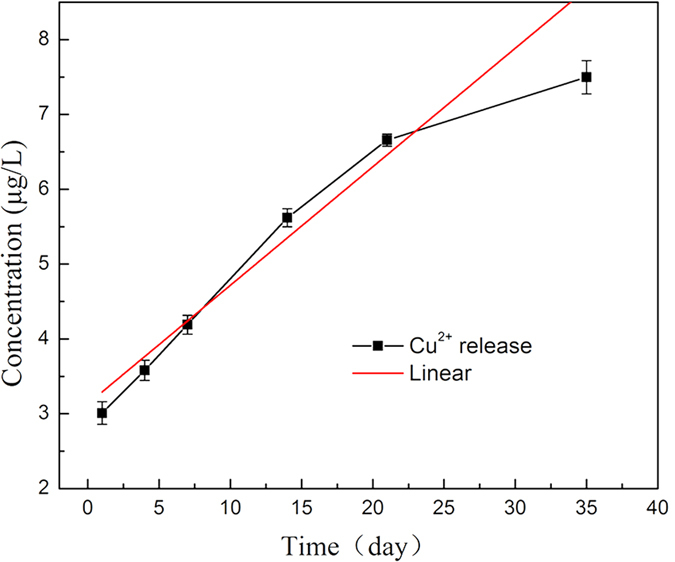
Cumulative Cu^2+^ concentration curve released from Ti-Cu alloy in 0.9% NaCl solution at 37 °C.

To further understand the copper ions release from the Ti-Cu alloy and their biological effects, the amount of Cu^2+^ released from the surface of Ti-Cu alloy was measured by ICP-MS. The concentrations of released Cu^2+^ at different time points are presented in Fig. 4. The findings showed that the concentration of Cu^2 +^ had a linear trend with a slope of 1.584 × 10^−4^ μg/ml/day. This slope was defined to be the average daily release rate of Cu^2+^.

### Cytotoxicity

**Figure 5:**
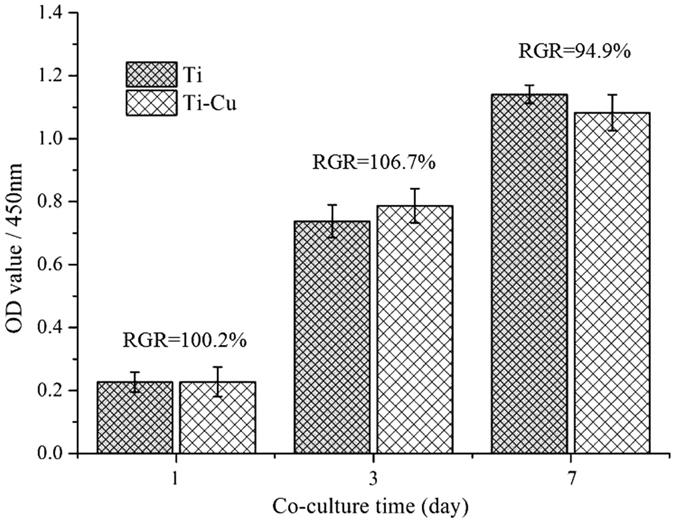
Viabilities of rBMSCs determined by measurement of the optical density (absorbance at 450 nm) in CCK-8 assay.

The proliferation and vitality of rat bone mesenchymal stem cells (rBMSCs) treated with Ti-Cu alloy were evaluated by the CCK-8 assay (Fig. 5), showing no significant difference in comparison with those of Ti. The relative growth rates (RGR) of rBMSCs on Ti-Cu alloy at 1, 3 and 7 days separately were 100.2%, 106.7% and 94.9%, which were demonstrated as non-cytotoxic according to the regulation in Table 1.

### Cell adhesion

**Figure 6:**
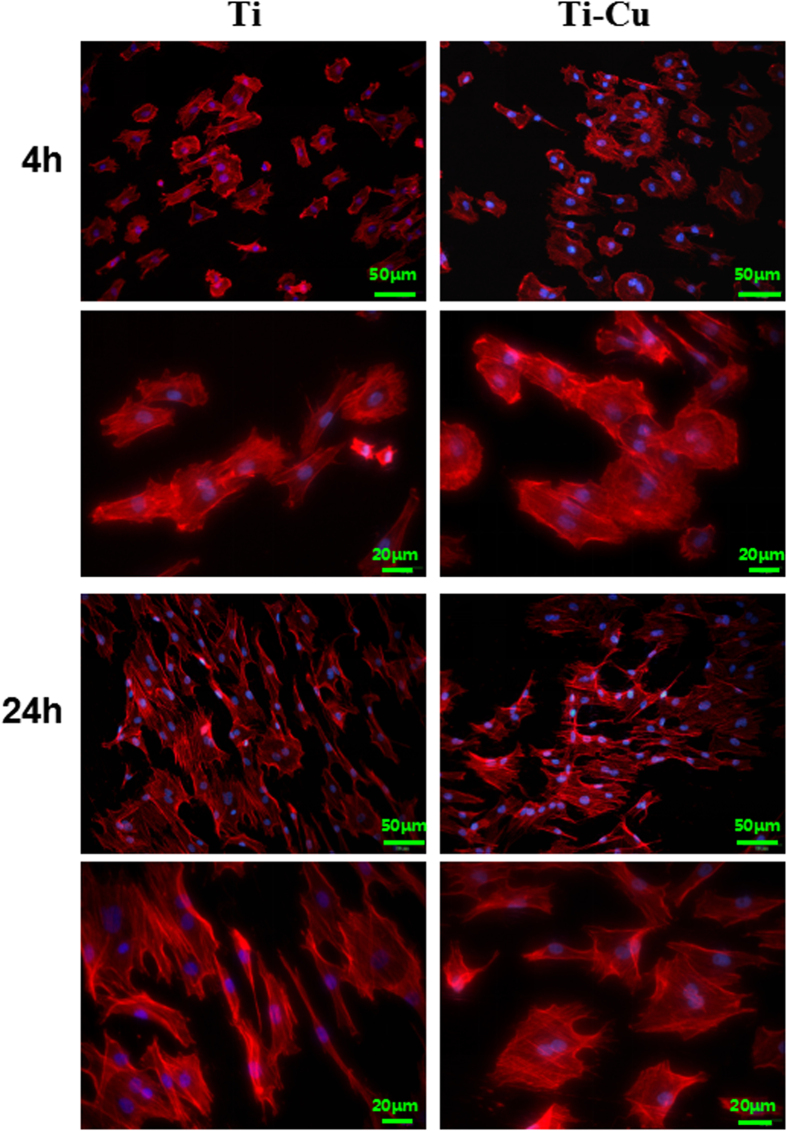
Fluorescent images of rBMSCs cultured on the surface of Ti and Ti-Cu alloy for 4 h and 24 h with actin stained with Rhodamine Phalloidin (red) and nuclei stained with DAPI (blue).

Mesenchymal stem cell adhesion was assayed by staining with rhodamine phalloidin and DAPI to visualize the F-actin (red) and nuclei (blue), as shown in Fig. 6. After culturing for 4 h, the expressions of F-actin on the surface of Ti-Cu alloy and Ti were found to be similar to each other, with more filopodia detected on the Ti-Cu alloy. In addition, the difference in the expression of F-actin after culturing for 24 h was not significant between two materials, and the cells on the surfaces exhibited a polygonal shape with a large number of filopodia and lamellipodia.

## Discussion

The formation of biofilms is a dynamic process that includes initial adhesion, aggregation and spread of bacteria. EPS secreted by the adhered bacteria gradually connects with the surrounding microorganisms and then embeds them, and finally a mature biofilm is formed^21^,^22^. Due to the protection of the biofilm, bacteria are afforded more protection^10^,^23^. Therefore, the inhibition of biofilm formation is regarded as the key for prevention of bacterial infection. Therefore, research on existing antimicrobial oral implant materials places emphasis on the inhibition of biofilm formation on the surface of materials. Mei *et al.* produced titania nanotubes (TiO_2_-NTs) containing Ag by utilizing a dual process encompassing anodization and silver plasma immersion ion implantation (Ag PIII), and demonstrated effects on *Porphyromonas gingivalis* (ATCC 33277) and *Actinobacillus actinomycetemcomitans* (ATCC 29523), effectively inhibiting the formation of biofilms, which would be expected to reduce the occurrence of peri-implantitis^13^. Likewise, Els Verraedt *et al.* used chlorhexidine as an oral implant coating on the surface of dental materials to inhibit biofilm formation^24^. In addition to this surface modification, bulk antimicrobial metallic material has also been developed recently, such as 304-Cu, 420-Cu and 317L-Cu antibacterial stainless steels, as well as a Ti-6Al-4V-xCu (x = 1, 3, 5) alloy, by addition of proper amount of Cu into currently used metallic materials. When in contact with the physiological environment, these Cu-bearing metallic biomaterials displayed strong antibacterial properties due to a continuous release of Cu ions into the surrounding environment^15^,^16^,^25^,^26^,^27^.

In order to develop a novel dental implant material with satisfactory antimicrobial properties which would discourage bacterial biofilm formation in the oral environment, the present study designed and fabricated a Cu-bearing Ti alloy by immobilizing appropriate amounts of Cu into pure Ti. The bactericidal effect of Cu^2+^ ions was confirmed by the quantitative results obtained against *S. mutans* and *P. gingivalis* using antibacterial tests (RT-PCR). Imaging analyzing (SEM, CLSM and DAPI) results further indicated that Ti-Cu alloy could inhibit biofilm formation. Immobilization of Cu into coatings or matrix material has been shown to provide antimicrobial activity against *Staphylococcus aureus* (*S.aureus)* and *Escherichia coli* (*E.coli*). Burghardtt *et al.* made implantation of a copper salt into a titanium implant material, and found that the growth of planktonic *S.aureus* was suppressed and adherent bacteria were cleared from the material surface within 24 h^28^. Wu *et al.* prepared Cu-containing mesoporous bioactive glass (Cu-MBG) scaffolds with interconnected large pores, which demonstrated antimicrobial properties^29^. Ren *et al.* prepared a Ti-6Al-4 V-5Cu alloy^19^,^30^, and demonstrated that Cu^2+^ ions was moved from the alloy surface to a free state, and the free Cu^2+^ ions could be bactericidal. However, the present work was the first to study Cu-bearing titanium with antimicrobial activity against species of bacteria existed in the oral environment.

**Figure 7:**
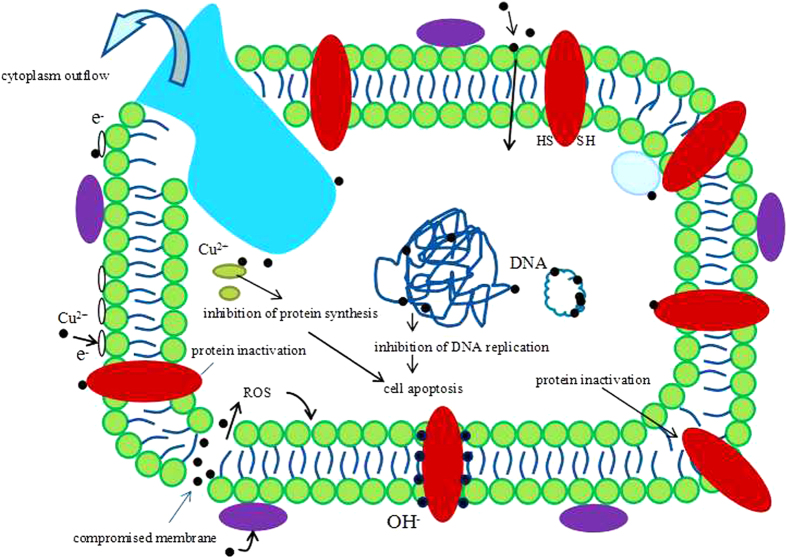
Schematic representation of the hypothetical scenario for the antibacterial mechanism of Cu^2+^ from Ti-Cu alloy.

The mechanism of antimicrobial activity of Ti-Cu was further investigated in this study. SEM and TEM were used to examine the treated cells of *S. mutans* and *P. gingivalis.* SEM and TEM observations in the present work and some qualitative findings suggest that direct interaction of the Ti-Cu alloy with the bacterial cell membrane may result in enhanced permeability of the membrane allowing entry of Cu ions into the cell^31^,^32^. The subsequent leakage of intracellular material due to membrane disruption may then cause shrinkage of the cell membrane, ultimately leading to cellular lysis^10^,^31^. Recently, Mei *et al.* found that Ti-6Al-4V-Cu alloy could disrupt the reactive oxygen species generation and the respiration of bacteria, and cause genetic toxicity by interfering with the replication of nuc (species-specific) and 16SrRNA genes. This may be the next step after cellular lysis as regards to the mechanism of antimicrobial activity of Cu^32^. Based upon the above analysis, a schematic representation of the hypothetical scenario for the antibacterial mechanism is shown in Fig. 7.

Though Ti-Cu alloy showed promising bactericidal properties, its biocompatibility must be carefully considered for its clinical applications. For the dissociated Cu ions, a high concentration has shown to possess superior sterilization ability, as found in the Bordeaux mixture. However, an overdose of Cu ions would be of low biocompatibility with subsequent harmful effect to human body. In addition to this, higher amount of Cu in the alloy will affect the plasticity, hot working performance and other properties. Thus, although a higher Cu dose would provide increased antibacterial efficacy, a balance between antimicrobial ability and other basic performance is necessary for selection of the optimum immobilization amount of Cu in the alloy. The minimum effective antibacterial concentration of Cu varies against different species of bacteria. Previous research^33^ has shown that the minimum inhibitory concentration (MIC) of Cu ion against *Staphylococcus aureus* was 448 μg/ml. Meanwhile, the MIC of Cu ion against *Escherichia coli* was 256 μg/ml. However, the average daily release amount of Cu^2+^ ions from the Ti-Cu alloy was measured to be 1.584 × 10^−4^ μg/ml, as shown in Fig. 4, which is much lower than the above MIC values, indicating that Ti-Cu alloy could possess highly efficient antimicrobial activity with such lower Cu^2+^ ions. Burghardt^28^ *et al.* reported that the threshold for toxic effect of copper ions on mesenchymal stem cells was 0.5 mM. Cao *et al.* demonstrated that the percentage relative growth rate (%RGR) for 72 h was 77.61% with 26.02 μg/ml Cu ions, which implies that this Cu dose has caused cytotoxicity according to ISO 10993–5 standard^34^,^35^. However, the average daily release amount of Cu ions from Ti-Cu alloy, 1.584 × 10^−4^ μg/ml, is much lower than the above amount. Considering that a Ti-Cu alloy implant is a cylinder with dimensions of 10 × 15 mm^3^, its maximum concentration of released Cu^2+^ ions could be about 0.014 μg/day, which is also far below the recommended daily adult intake of 2–3 mg by World Health Organization (WHO). Thus, the released amount of Cu^2+^ ions from Ti-Cu alloy would present no cytotoxicity as shown from the above data analysis. Furthermore, the result of the CCK-8 assay indicated the non-cytotoxic feature of the Ti-Cu alloy. Also, cells adhered onto the surface of the Ti-Cu alloy and demonstrated a normal cytoskeleton, which explains why Ti-Cu alloy was beneficial for the growth of rBMSCs. However, further *in vivo* studies should be conducted in order to confirm the present findings, aiming to develop a novel multi-functional titanium alloy for dental applications.

## Methods

### Sample preparation

A Ti-5wt.%Cu titanium alloy was fabricated by vacuum melting from high purity titanium (Ti) and Cu in a consumable electrode arc-melting furnace. The ingot was initially hot forged to bar with 40 mm diameter and then treated at 850 °C for 2 h followed by cooling in furnace. Ti-Cu alloy and commercially pure Ti were machined to small samples with diameter of 10 mm and thickness of 2 mm, polished and ultrasonically cleaned in acetone, ethanol, and sterile deionized water, and finally disinfected by ultraviolet light prior to experiments.

### Bacteria culture

*Streptococcus mutans* (*S. mutans* ATCC 25175, provided by the Laboratory Center, China Medical University, Shenyang) and *Porphyromonas gingivalis* (*P. gingivalis*, ATCC33277, provided by the Laboratory Center, China Medical University, Shenyang) were cultured on BHI-S blood agar plates (BHI, Oxoid, supplemented with 5 mg/ml yeast extract, 5 mg/ml hemin and 0.2 mg/ml menadione) under standard anaerobic conditions (80% N_2_, 10% H_2_ and 10% CO_2_, at 37 °C). Both species of bacteria were used in the various assays. The sterile samples were rinsed three times with sterile phosphate buffered saline (PBS) solution, then placed in 24-well culture plates, and separately incubated with 800 μl bacterial suspension for 24 h (1 × 10^5^ cfu/ml for *S. mutans*, 1 × 10^7^ cfu/ml for *P. gingivalis*).

### Quantitative (Real time) PCR

After incubation with test species for 24 h, the samples were vortexed three times with PBS (0.1 mol/l, pH 7.2) to collect the bacteria adherent to the surface, and the DNA was extracted using a Bacterial DNA extraction kit (Takara, Japan). The concentration and purity of DNA were measured by ELISA (Infinite 200 PRO, China) and then real time PCR was performed using a real time PCR system (ABI 7500 fast, US). Three-step PCR was performed at 50 °C for 2 min; 95 °C for 2 min; and 40 cycles at 95 °C for 15 s, 60 °C for 30 s and finally 72 °C for 1 min. Triplicate reactions were prepared with 20 μl of PCR mixture containing 10 μl of SYBR Premix Ex Taq (Tli RNaseH Plus), 1 μl of cDNA, 0.5 μl of PCR forward primer and 0.5 μl of PCR reverse primer, and 8 μl of sterile distilled water. *S. mutans’* glucosyltransferase-genes (*gtfB* and *gtfC*) and *P. gingivalis*’ normalizing internal standard gene (16S rRNA) were chosen as the primers as given in Table 2^ ^36,37,38,39. The real-time PCR produced a linear quantitative detection range over concentrations spanning seven exponential values, with a detection limit of a few copies of genomic DNA per reaction tube^39^. Three independent experiments were performed for each sample, and data analyses were conducted by using LightCycler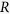 Software 3.5.

### DAPI

DAPI (4′,6-diamidino-2-phenylindole) is a cell permeable fluorescent minor groove-binding probe for DNA. It passes through the microbial cell membrane, and binds to double-stranded DNA to form a stable fluorescent complex. DAPI-DNA complex shows a light blue fluorescent color under excitation of UV-light. This study adopted the method of DAPI staining to observe the differences in quantity and distribution of bacteria on the surfaces of experimental and control materials. After culture with bacteria for 24 h, the samples were rinsed three times with PBS, stained with 1 μl DAPI for 15–30 min in darkness, and then rinsed three times with PBS again. Finally, the samples were observed under a fluorescence microscope (FM-600, China) and the images were obtained with a × 10 objective. Three independent experiments were performed.

### CLSM analysis of bacterial biofilm

Confocal laser scanning microscopy (CLSM) and a LIVE/DEAD Bacterial Viability Kit (Invitrogen Inc, USA) were used to study the viability of bacteria on the surface of the different samples. The green SYTO-9 probe labels live cells, whereas the red propidium iodide (PI) probe labels dead cells. After exposure to bacteria for 24 h, the biofilm formed on samples was rinsed three times with PBS, and stained with 200 μl of the staining solution comprised of 1.5 μl SYTO-9, 1.5 μl PI and 1 ml sterilized distilled water for 15 min in darkness. After rinsed three times with PBS, the biofilm was examined by CLSM (Olympus FV10-ASW, Japan) and the images were obtained with a × 20 objective. The images were then analyzed and three-dimension imaging reconstructed by using a NIS Viewer software. In this test, three independent biofilm analyses were performed.

### SEM observations

After incubation for 24 h, the formed biofilms were lightly rinsed three times with PBS, fixed with glutaraldehyde (2.5% v/v) at 4 °C for 4 h, washed in PBS, and then dehydrated in a series of ethanol solutions (50%, 70%, 95% and 100%) for 10 min each. The samples were then dried at room temperature and sputter-coated with gold. The surface morphology of the biofilms was observed on a scanning electron microscope (SEM, Phillips XL30FEG, Netherlands) at 12 kV in high-vacuum mode.

### TEM observations

TEM was performed to examine the intracellular changes in the two oral bacterial strains. After 24 h of co-culture, the formed biofilm on samples were washed twice with PBS and then centrifuged at 3000 rpm for 5 min. The pellets were separately fixed with a mixture (1: 1) of 2.5% v/v glutaraldehyde and 4% w/v paraformaldehyde at 4 °C overnight. The samples were then washed with PBS three times and the pellets were fixed in 1% (wt/vol) osmium tetroxide (OsO_4_) at room temperature for 2 h. Cells were then dehydrated with an ascending concentrations of ethyl alcohol (50%, 70%, 80%, 90%, 95% and 100%) for 15 min each. Dehydrated samples were then embedded in paraffin wax and thin sections (approximately 60 nm) were cut using an ultramicrotome (PowerTome-PC; RMC, America). These obtained sections were sequentially placed on the copper net and stained with 2% uranyl acetate and Reynolds’ lead citrate. Finally the samples were viewed on a transmission electron microscope (TEM, TECNAI G^2^, Electron Optics, America) at 100 kV in high-vacuum mode.

### Measurement of Cu^2+^ release

According to the ISO 10993-12 standard^40^, the Cu^2+^ released from Ti-Cu alloy for various periods of time was monitored in 0.9% NaCl solution at 37 °C. The concentrations of released Cu^2+^ of each harvested sample at 1, 4, 7, 14, 21 and 35 days were determined by inductively-coupled plasma mass spectrometry (ICP-MS, Thermo, America).

### Cytotoxicity

To evaluate cytotoxicity of the Ti-Cu alloy, according to the ISO 10993-5 standard^35^, a 0.5 ml rat bone mesenchymal stem cells (rBMSCs) suspension was seeded onto the samples at a density of 2 × 10^4^ cells/ml in 48-well tissue culture plates. Before seeding the cells, samples were sterilized with 75% alcohol for 2 h and rinsed twice with sterile PBS. A Cell Counting kit-8 (CCK-8) (Beyotime, China) was used to evaluate the cytotoxicity. After incubation periods of 1, 3, and 7 days, the samples were rinsed three times with sterile PBS and incubated with 250 μl MEM Alpha Modification (α-MEM, Hyclone, USA) containing 10% (v/v) CCK-8 solution at 37 °C for 3 h. 100 μl medium was taken from each well and transferred to a new 96-well plate and the optical density (OD) was determined by ELISA (Infinite 200 PRO, China) at 450 nm. The relative growth rate (RGR) of cells was calculated by Equation (1), and the RGR higher than 75% was considered as non-cytotoxic as shown in Table 1^ ^19.


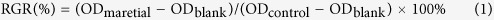


### Cell adhesion

The rBMSCs were seeded onto the samples at a density of 2 × 10^4^ cells/ml for 4 h and 24 h to allow cell attachment. After culturing, the samples were washed three times with sterile PBS, fixed with 4% paraformaldehyde (PFA) solution (Sigma, USA) for 5 min at room temperature, and then permeabilized with 0.1% (v/v) Triton X-100 (Amresco, USA) for 7-8 min. Afterwards, the cells attached on the samples were rinsed twice by sterile PBS and stained with Rhodamine Phalloidin (Sigma, USA) at room temperature in darkness for 40 min and further stained with DAPI (Sigma, USA) for 5 min. The F-actin and cell nuclei were examined by a fluorescence microscope (FM-600, China).

### Statistical analysis

All the experiments were carried out in triplicate. For each set, the relevant data was summarized as the mean standard deviation. Statistical significance was determined using SPSS 13.0 (SPSS Inc., Chicago, IL). The results were considered statistically significant at *P ≤ 0.05, **P ≤ 0.01 and ***P ≤ 0.001.

## Conclusion

The newly developed Ti-Cu alloy was shown to provide an effective and sustainable bactericidal effect against the oral bacterial species of *S. mutans* and *P. gingivalis*. This alloy also inhibited bacterial adhesion and biofilm formation. It was also shown to be biocompatible with only minimal Cu^2+^ release, which is substantially below the recommended daily intake of Cu. Based upon the present findings it can be deduced that the Ti-Cu alloy is suitable as a dental implant material that is both highly antimicrobial and biocompatible, making it a desirable material for future clinical investigations.

## Additional Information

**How to cite this article**: Liu, R. *et al.* Antibacterial effect of copper-bearing titanium alloy (Ti-Cu) against *Streptococcus mutans* and *Porphyromonas gingivalis. Sci. Rep.* **6**, 29985; doi: 10.1038/srep29985 (2016).

## Figures and Tables

**Table 1:** Classification standard of cell toxicity.

RGR/%	Grade	Evaluation criterion	RGR/%	Grade	Evaluation criterion
≥100	0	Non-cytotoxic	25–49	3	Cytotoxic
75–99	1	Non-cytotoxic	1–24	4	Cytotoxic
50–74	2	—	0	5	Cytotoxic

**Table 2:** Nucleotide sequences of primers used in this study.

Strains	Gene Name	Primer Sequence(5′-3′)
*S. mutansATCC 25175*	*gtfB* and *gtfC*	F: AGCCATGCGCAATCAACAGGTT
		R: CGCAACGCGAACATCTTGATTAG
*P. gingivalisATCC 33277*	16s rRNA	F: AGGCAGCTTGCCATACTGCG
		R: ACTGTTAGCAACTACCGATGT
